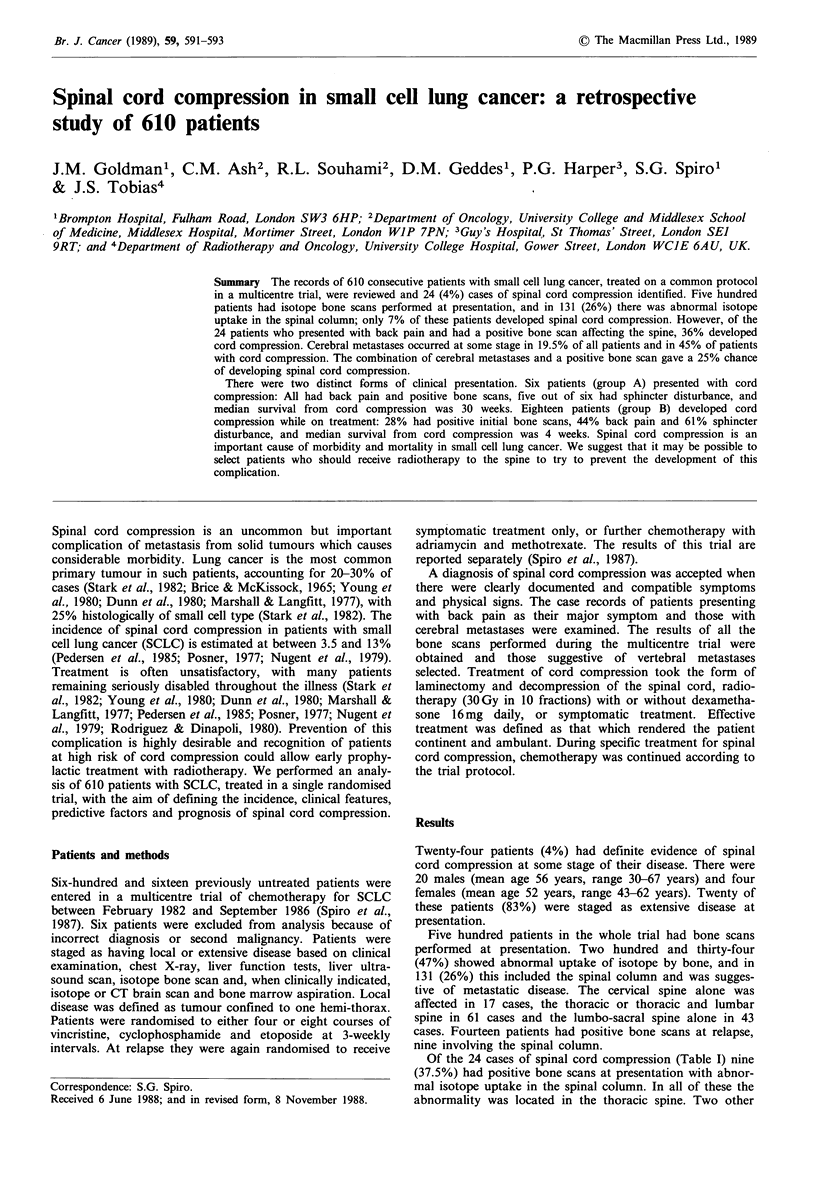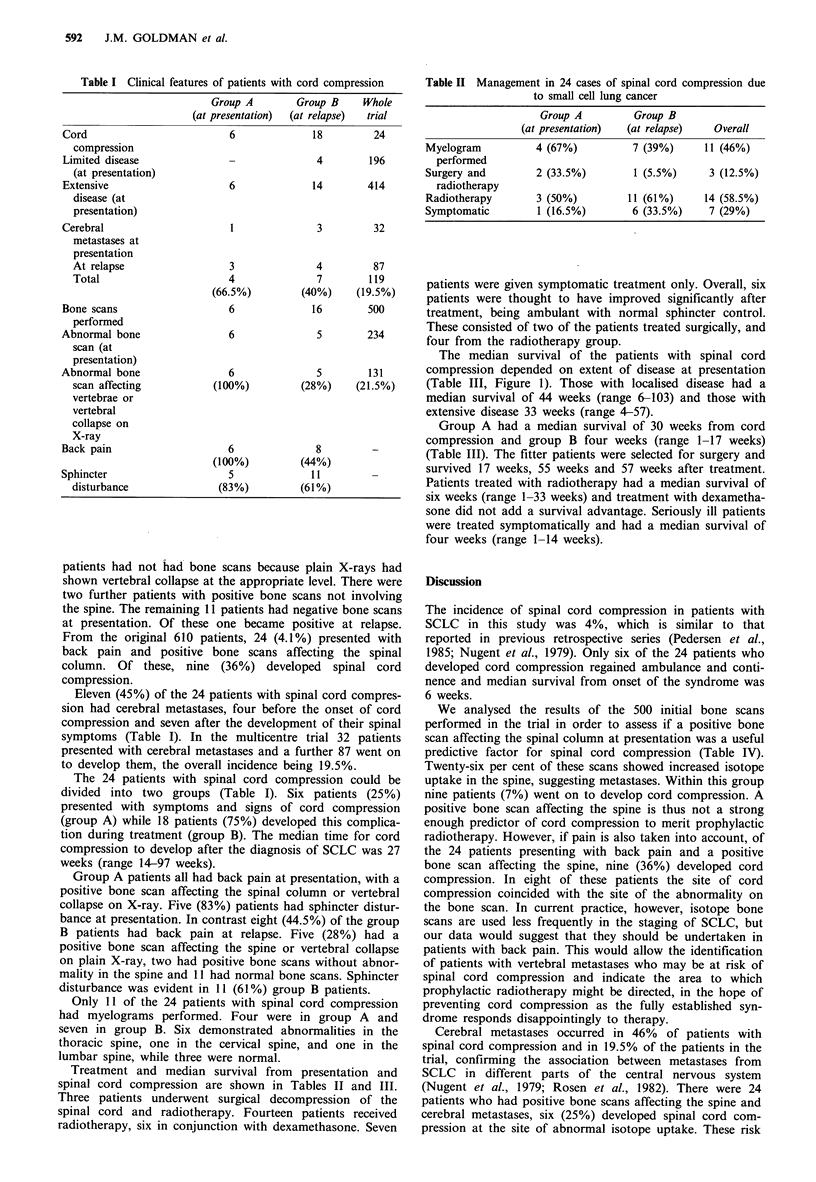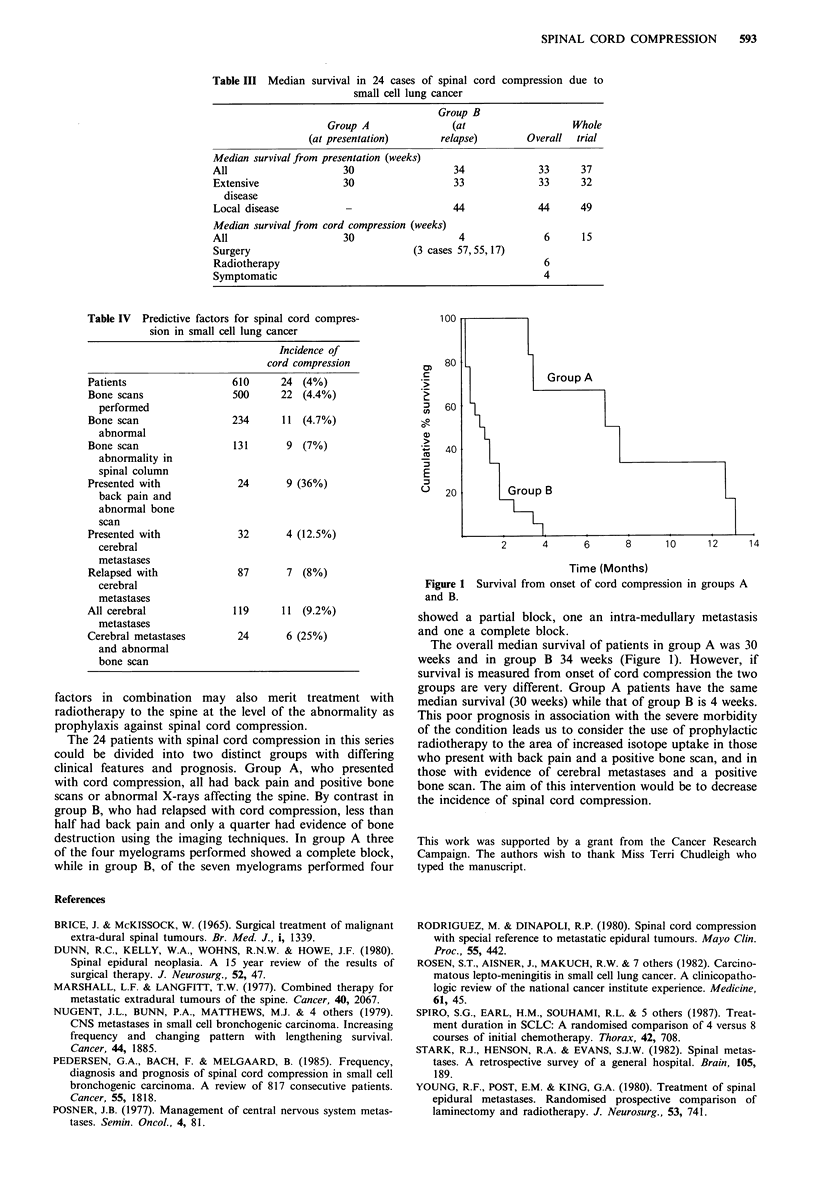# Spinal cord compression in small cell lung cancer: a retrospective study of 610 patients.

**DOI:** 10.1038/bjc.1989.119

**Published:** 1989-04

**Authors:** J. M. Goldman, C. M. Ash, R. L. Souhami, D. M. Geddes, P. G. Harper, S. G. Spiro, J. S. Tobias

**Affiliations:** Brompton Hospital, London, UK.

## Abstract

The records of 610 consecutive patients with small cell lung cancer, treated on a common protocol in a multicentre trial, were reviewed and 24 (4%) cases of spinal cord compression identified. Five hundred patients had isotope bone scans performed at presentation, and in 131 (26%) there was abnormal isotope uptake in the spinal column; only 7% of these patients developed spinal cord compression. However, of the 24 patients who presented with back pain and had a positive bone scan affecting the spine, 36% developed cord compression. Cerebral metastases occurred at some stage in 19.5% of all patients and in 45% of patients with cord compression. The combination of cerebral metastases and a positive bone scan gave a 25% chance of developing spinal cord compression. There were two distinct forms of clinical presentation. Six patients (group A) presented with cord compression: All had back pain and positive bone scans, five out of six had sphincter disturbance, and median survival from cord compression was 30 weeks. Eighteen patients (group B) developed cord compression while on treatment: 28% had positive initial bone scans, 44% back pain and 61% sphincter disturbance, and median survival from cord compression was 4 weeks. Spinal cord compression is an important cause of morbidity and mortality in small cell lung cancer. We suggest that it may be possible to select patients who should receive radiotherapy to the spine to try to prevent the development of this complication.


*Br. J. Cancer* (1989), **59**, 591–593 © The Macmillan Press Ltd., 1989

### Spinal cord compression in small cell lung cancer: a retrospective study of 610 patients

J.M. Goldman[1], C.M. Ash[2], R.L. Souhami[2], D.M. Geddes[1], P.G. Harper[3], S.G. Spiro[1] & J.S. Tobias[4]

[1]*Brompton Hospital, Fulham Road, London SW3 6HP;* [2]*Department of Oncology, University College and Middlesex School of Medicine, Middlesex Hospital, Mortimer Street, London W1P 7PN;* [3]*Guy's Hospital, St Thomas' Street, London SE1 9RT; and* [4]*Department of Radiotherapy and Oncology, University College Hospital, Gower Street, London WC1E 6AU, UK.*

**Summary** The records of 610 consecutive patients with small cell lung cancer, treated on a common protocol in a multicentre trial, were reviewed and 24 (4%) cases of spinal cord compression identified. Five hundred patients had isotope bone scans performed at presentation, and in 131 (26%) there was abnormal isotope uptake in the spinal column; only 7% of these patients developed spinal cord compression. However, of the 24 patients who presented with back pain and had a positive bone scan affecting the spine, 36% developed cord compression. Cerebral metastases occurred at some stage in 19.5% of all patients and in 45% of patients with cord compression. The combination of cerebral metastases and a positive bone scan gave a 25% chance of developing spinal cord compression.

There were two distinct forms of clinical presentation. Six patients (group A) presented with cord compression: All had back pain and positive bone scans, five out of six had sphincter disturbance, and median survival from cord compression was 30 weeks. Eighteen patients (group B) developed cord compression while on treatment: 28% had positive initial bone scans, 44% back pain and 61% sphincter disturbance, and median survival from cord compression was 4 weeks. Spinal cord compression is an important cause of morbidity and mortality in small cell lung cancer. We suggest that it may be possible to select patients who should receive radiotherapy to the spine to try to prevent the development of this complication.

Spinal cord compression is an uncommon but important complication of metastasis from solid tumours which causes considerable morbidity. Lung cancer is the most common primary tumour in such patients, accounting for 20–30% of cases (Stark *et al.*, 1982; Brice & McKissock, 1965; Young *et al.*, 1980; Dunn *et al.*, 1980; Marshall & Langfitt, 1977), with 25% histologically of small cell type (Stark *et al.*, 1982). The incidence of spinal cord compression in patients with small cell lung cancer (SCLC) is estimated at between 3.5 and 13% (Pedersen *et al.*, 1985; Posner, 1977; Nugent *et al.*, 1979). Treatment is often unsatisfactory, with many patients remaining seriously disabled throughout the illness (Stark *et al.*, 1982; Young *et al.*, 1980; Dunn *et al.*, 1980; Marshall & Langfitt, 1977; Pedersen *et al.*, 1985; Posner, 1977; Nugent *et al.*, 1979; Rodriguez & Dinapoli, 1980). Prevention of this complication is highly desirable and recognition of patients at high risk of cord compression could allow early prophylactic treatment with radiotherapy. We performed an analysis of 610 patients with SCLC, treated in a single randomised trial, with the aim of defining the incidence, clinical features, predictive factors and prognosis of spinal cord compression.

### Patients and methods

Six-hundred and sixteen previously untreated patients were entered in a multicentre trial of chemotherapy for SCLC between February 1982 and September 1986 (Spiro *et al.*, 1987). Six patients were excluded from analysis because of incorrect diagnosis or second malignancy. Patients were staged as having local or extensive disease based on clinical examination, chest X-ray, liver function tests, liver ultrasound scan, isotope bone scan and, when clinically indicated, isotope or CT brain scan and bone marrow aspiration. Local disease was defined as tumour confined to one hemi-thorax. Patients were randomised to either four or eight courses of vincristine, cyclophosphamide and etoposide at 3-weekly intervals. At relapse they were again randomised to receive symptomatic treatment only, or further chemotherapy with adriamycin and methotrexate. The results of this trial are reported separately (Spiro *et al.*, 1987).

A diagnosis of spinal cord compression was accepted when there were clearly documented and compatible symptoms and physical signs. The case records of patients presenting with back pain as their major symptom and those with cerebral metastases were examined. The results of all the bone scans performed during the multicentre trial were obtained and those suggestive of vertebral metastases selected. Treatment of cord compression took the form of laminectomy and decompression of the spinal cord, radiotherapy (30 Gy in 10 fractions) with or without dexamethasone 16 mg daily, or symptomatic treatment. Effective treatment was defined as that which rendered the patient continent and ambulant. During specific treatment for spinal cord compression, chemotherapy was continued according to the trial protocol.

### Results

Twenty-four patients (4%) had definite evidence of spinal cord compression at some stage of their disease. There were 20 males (mean age 56 years, range 30–67 years) and four females (mean age 52 years, range 43–62 years). Twenty of these patients (83%) were staged as extensive disease at presentation.

Five hundred patients in the whole trial had bone scans performed at presentation. Two hundred and thirty-four (47%) showed abnormal uptake of isotope by bone, and in 131 (26%) this included the spinal column and was suggestive of metastatic disease. The cervical spine alone was affected in 17 cases, the thoracic or thoracic and lumbar spine in 61 cases and the lumbo-sacral spine alone in 43 cases. Fourteen patients had positive bone scans at relapse, nine involving the spinal column.

Of the 24 cases of spinal cord compression (Table I) nine (37.5%) had positive bone scans at presentation with abnormal isotope uptake in the spinal column. In all of these the abnormality was located in the thoracic spine. Two other

Correspondence: S.G. Spiro.
Received 6 June 1988; and in revised form, 8 November 1988.

**Table I** Clinical features of patients with cord compression

| | Group A (at presentation) | Group B (at relapse) | Whole trial |
|---|---|---|---|
| Cord compression | 6 | 18 | 24 |
| Limited disease (at presentation) | – | 4 | 196 |
| Extensive disease (at presentation) | 6 | 14 | 414 |
| Cerebral metastases at presentation | 1 | 3 | 32 |
| At relapse | 3 | 4 | 87 |
| Total | 4 (66.5%) | 7 (40%) | 119 (19.5%) |
| Bone scans performed | 6 | 16 | 500 |
| Abnormal bone scan (at presentation) | 6 | 5 | 234 |
| Abnormal bone scan affecting vertebrae or vertebral collapse on X-ray | 6 (100%) | 5 (28%) | 131 (21.5%) |
| Back pain | 6 (100%) | 8 (44%) | – |
| Sphincter disturbance | 5 (83%) | 11 (61%) | – |

**Table II** Management in 24 cases of spinal cord compression due to small cell lung cancer

| | Group A (at presentation) | Group B (at relapse) | Overall |
|---|---|---|---|
| Myelogram performed | 4 (67%) | 7 (39%) | 11 (46%) |
| Surgery and radiotherapy | 2 (33.5%) | 1 (5.5%) | 3 (12.5%) |
| Radiotherapy | 3 (50%) | 11 (61%) | 14 (58.5%) |
| Symptomatic | 1 (16.5%) | 6 (33.5%) | 7 (29%) |

patients had not had bone scans because plain X-rays had shown vertebral collapse at the appropriate level. There were two further patients with positive bone scans not involving the spine. The remaining 11 patients had negative bone scans at presentation. Of these one became positive at relapse. From the original 610 patients, 24 (4.1%) presented with back pain and positive bone scans affecting the spinal column. Of these, nine (36%) developed spinal cord compression.

Eleven (45%) of the 24 patients with spinal cord compression had cerebral metastases, four before the onset of cord compression and seven after the development of their spinal symptoms (Table I). In the multicentre trial 32 patients presented with cerebral metastases and a further 87 went on to develop them, the overall incidence being 19.5%.

The 24 patients with spinal cord compression could be divided into two groups (Table I). Six patients (25%) presented with symptoms and signs of cord compression (group A) while 18 patients (75%) developed this complication during treatment (group B). The median time for cord compression to develop after the diagnosis of SCLC was 27 weeks (range 14–97 weeks).

Group A patients all had back pain at presentation, with a positive bone scan affecting the spinal column or vertebral collapse on X-ray. Five (83%) patients had sphincter disturbance at presentation. In contrast eight (44.5%) of the group B patients had back pain at relapse. Five (28%) had a positive bone scan affecting the spine or vertebral collapse on plain X-ray, two had positive bone scans without abnormality in the spine and 11 had normal bone scans. Sphincter disturbance was evident in 11 (61%) group B patients.

Only 11 of the 24 patients with spinal cord compression had myelograms performed. Four were in group A and seven in group B. Six demonstrated abnormalities in the thoracic spine, one in the cervical spine, and one in the lumbar spine, while three were normal.

Treatment and median survival from presentation and spinal cord compression are shown in Tables II and III. Three patients underwent surgical decompression of the spinal cord and radiotherapy. Fourteen patients received radiotherapy, six in conjunction with dexamethasone. Seven patients were given symptomatic treatment only. Overall, six patients were thought to have improved significantly after treatment, being ambulant with normal sphincter control. These consisted of two of the patients treated surgically, and four from the radiotherapy group.

The median survival of the patients with spinal cord compression depended on extent of disease at presentation (Table III, Figure 1). Those with localised disease had a median survival of 44 weeks (range 6–103) and those with extensive disease 33 weeks (range 4–57).

Group A had a median survival of 30 weeks from cord compression and group B four weeks (range 1–17 weeks) (Table III). The fitter patients were selected for surgery and survived 17 weeks, 55 weeks and 57 weeks after treatment. Patients treated with radiotherapy had a median survival of six weeks (range 1–33 weeks) and treatment with dexamethasone did not add a survival advantage. Seriously ill patients were treated symptomatically and had a median survival of four weeks (range 1–14 weeks).

### Discussion

The incidence of spinal cord compression in patients with SCLC in this study was 4%, which is similar to that reported in previous retrospective series (Pedersen *et al.*, 1985; Nugent *et al.*, 1979). Only six of the 24 patients who developed cord compression regained ambulance and continence and median survival from onset of the syndrome was 6 weeks.

We analysed the results of the 500 initial bone scans performed in the trial in order to assess if a positive bone scan affecting the spinal column at presentation was a useful predictive factor for spinal cord compression (Table IV). Twenty-six per cent of these scans showed increased isotope uptake in the spine, suggesting metastases. Within this group nine patients (7%) went on to develop cord compression. A positive bone scan affecting the spine is thus not a strong enough predictor of cord compression to merit prophylactic radiotherapy. However, if pain is also taken into account, of the 24 patients presenting with back pain and a positive bone scan affecting the spine, nine (36%) developed cord compression. In eight of these patients the site of cord compression coincided with the site of the abnormality on the bone scan. In current practice, however, isotope bone scans are used less frequently in the staging of SCLC, but our data would suggest that they should be undertaken in patients with back pain. This would allow the identification of patients with vertebral metastases who may be at risk of spinal cord compression and indicate the area to which prophylactic radiotherapy might be directed, in the hope of preventing cord compression as the fully established syndrome responds disappointingly to therapy.

Cerebral metastases occurred in 46% of patients with spinal cord compression and in 19.5% of the patients in the trial, confirming the association between metastases from SCLC in different parts of the central nervous system (Nugent *et al.*, 1979; Rosen *et al.*, 1982). There were 24 patients who had positive bone scans affecting the spine and cerebral metastases, six (25%) developed spinal cord compression at the site of abnormal isotope uptake. These risk

**Table III** Median survival in 24 cases of spinal cord compression due to small cell lung cancer

| | *Group A* (*at presentation*) | *Group B* (*at relapse*) | *Overall* | *Whole trial* |
|---|---|---|---|---|
| *Median survival from presentation* (*weeks*) | | | | |
| All | 30 | 34 | 33 | 37 |
| Extensive disease | 30 | 33 | 33 | 32 |
| Local disease | – | 44 | 44 | 49 |
| *Median survival from cord compression* (*weeks*) | | | | |
| All | 30 | 4 | 6 | 15 |
| Surgery | | (3 cases 57,55,17) | | |
| Radiotherapy | | | 6 | |
| Symptomatic | | | 4 | |

**Table IV** Predictive factors for spinal cord compression in small cell lung cancer

| | | *Incidence of cord compression* |
|---|---|---|
| Patients | 610 | 24 (4%) |
| Bone scans performed | 500 | 22 (4.4%) |
| Bone scan abnormal | 234 | 11 (4.7%) |
| Bone scan abnormality in spinal column | 131 | 9 (7%) |
| Presented with back pain and abnormal bone scan | 24 | 9 (36%) |
| Presented with cerebral metastases | 32 | 4 (12.5%) |
| Relapsed with cerebral metastases | 87 | 7 (8%) |
| All cerebral metastases | 119 | 11 (9.2%) |
| Cerebral metastases and abnormal bone scan | 24 | 6 (25%) |

factors in combination may also merit treatment with radiotherapy to the spine at the level of the abnormality as prophylaxis against spinal cord compression.

The 24 patients with spinal cord compression in this series could be divided into two distinct groups with differing clinical features and prognosis. Group A, who presented with cord compression, all had back pain and positive bone scans or abnormal X-rays affecting the spine. By contrast in group B, who had relapsed with cord compression, less than half had back pain and only a quarter had evidence of bone destruction using the imaging techniques. In group A three of the four myelograms performed showed a complete block, while in group B, of the seven myelograms performed four

**Figure 1** Survival from onset of cord compression in groups A and B.

showed a partial block, one an intra-medullary metastasis and one a complete block.

The overall median survival of patients in group A was 30 weeks and in group B 34 weeks (Figure 1). However, if survival is measured from onset of cord compression the two groups are very different. Group A patients have the same median survival (30 weeks) while that of group B is 4 weeks. This poor prognosis in association with the severe morbidity of the condition leads us to consider the use of prophylactic radiotherapy to the area of increased isotope uptake in those who present with back pain and a positive bone scan, and in those with evidence of cerebral metastases and a positive bone scan. The aim of this intervention would be to decrease the incidence of spinal cord compression.

This work was supported by a grant from the Cancer Research Campaign. The authors wish to thank Miss Terri Chudleigh who typed the manuscript.

### References

BRICE, J. & McKISSOCK, W. (1965). Surgical treatment of malignant extra-dural spinal tumours. *Br. Med. J.*, **i**, 1339.

DUNN, R.C., KELLY, W.A., WOHNS, R.N.W. & HOWE, J.F. (1980). Spinal epidural neoplasia. A 15 year review of the results of surgical therapy. *J. Neurosurg.*, **52**, 47.

MARSHALL, L.F. & LANGFITT, T.W. (1977). Combined therapy for metastatic extradural tumours of the spine. *Cancer*, **40**, 2067.

NUGENT, J.L., BUNN, P.A., MATTHEWS, M.J. & 4 others (1979). CNS metastases in small cell bronchogenic carcinoma. Increasing frequency and changing pattern with lengthening survival. *Cancer*, **44**, 1885.

PEDERSEN, G.A., BACH, F. & MELGAARD, B. (1985). Frequency, diagnosis and prognosis of spinal cord compression in small cell bronchogenic carcinoma. A review of 817 consecutive patients. *Cancer*, **55**, 1818.

POSNER, J.B. (1977). Management of central nervous system metastases. *Semin. Oncol.*, **4**, 81.

RODRIGUEZ, M. & DINAPOLI, R.P. (1980). Spinal cord compression with special reference to metastatic epidural tumours. *Mayo Clin. Proc.*, **55**, 442.

ROSEN, S.T., AISNER, J., MAKUCH, R.W. & 7 others (1982). Carcinomatous lepto-meningitis in small cell lung cancer. A clinicopathologic review of the national cancer institute experience. *Medicine*, **61**, 45.

SPIRO, S.G., EARL, H.M., SOUHAMI, R.L. & 5 others (1987). Treatment duration in SCLC: A randomised comparison of 4 versus 8 courses of initial chemotherapy. *Thorax*, **42**, 708.

STARK, R.J., HENSON, R.A. & EVANS, S.J.W. (1982). Spinal metastases. A retrospective survey of a general hospital. *Brain*, **105**, 189.

YOUNG, R.F., POST, E.M. & KING, G.A. (1980). Treatment of spinal epidural metastases. Randomised prospective comparison of laminectomy and radiotherapy. *J. Neurosurg.*, **53**, 741.